# Supplementary material for: MetaRibo-Seq measures translation in microbiomes
Source: Nat Commun. 2020 Jun 29;11:3268. doi: 10.1038/s41467-020-17081-z (PMC7324362; doi:10.1038/s41467-020-17081-z)
Supplement: Supplementary file 10 — Supplementary Data 7 [file 41467_2020_17081_MOESM10_ESM.zip › File2/Confidence_High_Taxonomy/227513_out.krona.html]

Javascript must be enabled to view this page.

members
magnitude
magnitudeUnassigned
count
unassigned
taxon
rank

227513\_out

3


SRS019327\_contig\_number\_contig-100\_5286.5286.5286
1

superkingdom
2
2

phylum
1239
2

class
186801
2

186802
2
order

family
1
186806

genus
1
1730


SRS050941\_contig\_number\_13061
species
1
1262887

family
186803
1

genus
43996
1

43997
1
species

SRS076764\_contig\_number\_contig-100\_1547.1548
